# Translational Research on Solid Tumors: Bridging Molecular Insights and Clinical Impact

**DOI:** 10.3390/cells14231918

**Published:** 2025-12-03

**Authors:** Milena Urbini, Paola Ulivi, Giorgia Marisi

**Affiliations:** Biosciences Laboratory, IRCCS Istituto Romagnolo per lo Studio dei Tumori (IRST) “Dino Amadori”, 47014 Meldola, FC, Italy; milena.urbini@irst.emr.it (M.U.); giorgia.marisi@irst.emr.it (G.M.)

## 1. Introduction

Translational oncology continues to advance through the integration of biological discoveries, technological innovations and clinical applications [1]. Its primary goal is to convert molecular insights into diagnostic, prognostic and therapeutic strategies that contribute to improved patient outcomes. The field has expanded rapidly with the development of high-throughput sequencing, digital pathology, advanced imaging, and computational modeling, allowing tumors to be characterized with an unprecedented level of detail. These developments also highlight the need for harmonized analytical frameworks, robust validation, and effective clinical implementation.

This Special Issue of *Cells* highlights how multidisciplinary approaches, spanning genomics, epigenetics, cellular imaging, and therapeutic innovation, are accelerating our understanding and treatment of solid tumors.

## 2. An Overview of Published Articles

### 2.1. Biomarkers for Precision Medicine

Tumor molecular characteristics, along with other patient-specific factors, strongly influence both prognosis and therapeutic response. In recent years, treatment personalization has become increasingly widespread, and we are approaching an era of true precision medicine for solid tumors. Several of the papers within this Special Issue describe newly identified prognostic biomarkers that may support therapy selection, as well as studies of novel targeted drugs which have been evaluated in in vitro models to provide a rationale for future therapeutic strategies.

In their review, Ladel et al. (Contribution 1) highlight the potential of epigenetic mechanisms to drive biomarker development and therapeutic innovation in appendiceal neoplasms, a rare and understudied group of malignancies. Similarly, Koll et al. (Contribution 2) demonstrate that KMT9α, a histone methyltransferase, acts as an epigenetic regulator of carcinogenesis and is highly expressed in basal-like muscle-invasive bladder cancer, particularly in tumors with squamous features. Interestingly, they found that nucleolar expression of KMT9α correlates with poor survival. Taken together, these findings support its potential as a therapeutic target in bladder cancer

In the realm of immunotherapy, Li et al. (Contribution 3) identified RELN mutations as potential predictors of improved efficacy of immune checkpoint inhibitors in melanoma and non-small cell lung cancer. They showed associations with better survival, higher response rates, and a more favorable immune microenvironment. These findings support the value of biomarker-based patient selection in immunotherapy.

Finally, the translation of novel drug design from chemistry to clinical relevance was exemplified by Facchin et al. (Contribution 4), who investigated the efficacy of ZR2002, a dual EGFR–DNA-targeting combi-molecule, in an osteosarcoma xenograft model. Their results highlight the therapeutic potential of rationally designed agents that couple receptor inhibition with direct DNA damage, offering an elegant paradigm for overcoming resistance mechanisms in solid tumors.

### 2.2. Circulating Tumor Cells

Liquid biopsy techniques have developed substantially over the past decade, and it has evolved from a promising research tool to a clinically relevant approach for real-time tumor assessment. Its strength lies in its ability to capture tumor-derived material from blood or other body fluids, providing a minimally invasive strategy suitable for serial monitoring. Broader analyses of circulating nucleic acids and proteins have shown encouraging results in early detection, diagnosis, and disease monitoring [2]. Among these approaches, the study of circulating tumor cells (CTCs) represents a key topic for cancer detection and characterization, offering a cellular perspective that complements nucleic acid-based analyses [3].

Advances in enrichment and characterization technologies have improved sensitivity, enabling more reliable longitudinal monitoring [3,4]. In their study, Magri et al. demonstrate that longitudinal CTC trajectories outperform single time-point measurements for prognostic assessment in metastatic colorectal cancer (Contribution 5).

Meanwhile, in their work, Buzalewicz et al. (Contribution 6) present an innovative tool with the potential to improve CTC detection. The authors show that quantitative phase imaging can visualize hypoxia-induced morphological changes in both normal and neoplastic colonic epithelial cells. Their research highlights how digital holotomography can detect optical and morphological alterations driven by hypoxia in colorectal cancer cells, underscoring the potential of advanced imaging techniques as translational tools for early CTC identification. Further studies which apply this method in the context of liquid biopsy are needed to validate these findings.

Together, these findings illustrate how CTCs can serve as prognostic indicators, highlight the growing importance of temporal biomarkers in understanding disease dynamics, and demonstrate the continuous evolution of various technologies that are providing increasingly sensitive and integrated approaches to meet the needs of translational research.

## 3. Future Perspectives

Tissue-based molecular profiling remains central to translational oncology, providing direct access to the genomic and epigenomic features that define tumor biology. However, recently, the field of translational oncology has gravitated toward integrated frameworks that combine tissue-derived molecular information, circulating biomarkers, imaging-derived technologies and clinical data.

The complementary nature of tissue-based profiling and liquid biopsy will become more central in future study designs, enabling improved detection of resistant clones, refined patient selection for targeted and immune therapies, and earlier identification of progression or relapse. Functional imaging will continue to enrich molecular interpretations by capturing real-time microenvironmental states. Each of these layers contributes distinct and complementary information and their integration is important for capturing tumor heterogeneity and the complexity of oncogenic processes with greater accuracy [5]. As analytical depth expands, standardized workflows and computational strategies capable of harmonizing heterogeneous datasets will become essential. Artificial intelligence and multimodal machine learning approaches are expected to play critical roles in connecting molecular alterations, imaging features and clinical trajectories, as highlighted in recent methodological studies [6]. Continued methodological refinement, collaborative research, and rigorous validation will be critical for translating these advancements into clinically meaningful applications.

## Abbreviations

The following abbreviations are used in this manuscript:

CTCs	Circulating tumor cells.
EGFR	Epidermal growth factor receptor.
KMT9α	Lysine methyl transferase 9.
RELN	Reelin.
